# Excessive processing and acetylation of OPA1 aggravate age‐related hearing loss via the dysregulation of mitochondrial dynamics

**DOI:** 10.1111/acel.14091

**Published:** 2024-01-24

**Authors:** Andi Zhang, Yi Pan, Hao Wang, Rui Ding, Tianyuan Zou, Dongye Guo, Yilin Shen, Peilin Ji, Weiyi Huang, Qing Wen, Quan Wang, Haixia Hu, Jichang Wu, Mingliang Xiang, Bin Ye

**Affiliations:** ^1^ Department of Otolaryngology & Head and Neck Surgery, Ruijin Hospital Shanghai Jiao Tong University School of Medicine Shanghai China; ^2^ Shanghai Key Laboratory of Translational Medicine on Ear and Nose Diseases Shanghai China; ^3^ Ear Institute Shanghai Jiao Tong University School of Medicine Shanghai China; ^4^ Department of Audiology & Speech‐Language Pathology, College of Health Science and Technology Shanghai Jiao Tong University School of Medicine Shanghai China

**Keywords:** age‐related hearing loss, hair cells, mitochondrial dynamics, OPA1, pyroptosis, SIRT3

## Abstract

The pathogenesis of age‐related hearing loss (ARHL) remains unclear. OPA1 is the sole fusion protein currently known to be situated in the inner mitochondrial membrane, which is pivotal for maintaining normal mitochondrial function. While it has already been demonstrated that mutations in OPA1 may lead to hereditary deafness, its involvement in the occurrence and development of ARHL has not been previously explored. In our study, we constructed D‐gal‐induced senescent HEI‐OC1 cells and the cochlea of C57BL/6J mice with a mutated SUMOylation site of SIRT3 using CRISPR/Cas9 technology. We found enhanced L‐OPA1 processing mediated by activated OMA1, and increased OPA1 acetylation resulting from reductions in SIRT3 levels in senescent HEI‐OC1 cells. Consequently, the fusion function of OPA1 was inhibited, leading to mitochondrial fission and pyroptosis in hair cells, ultimately exacerbating the aging process of hair cells. Our results suggest that the dysregulation of mitochondrial dynamics in cochlear hair cells in aged mice can be ameliorated by activating the SIRT3/OPA1 signaling. This has the potential to alleviate the senescence of cochlear hair cells and reduce hearing loss in mice. Our study highlights the significant roles played by the quantities of long and short chains and the acetylation activity of OPA1 in the occurrence and development of ARHL. This finding offers new perspectives and potential targets for the prevention and treatment of ARHL.

## Graphical abstract

The pivotal role of OPA1‐mediated excessive mitochondrial fission plays in the occurrence and development of age‐related hearing loss.

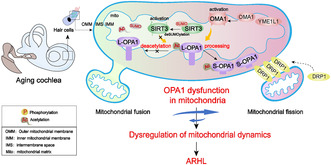

**Abbreviations**
ABR: auditory brainstem response
ARHL: age‐related hearing loss
ATP: adenosine triphosphate
Cas9: CRISPR‐associated protein 9
CRISPR: clustered regularly interspaced short palindromic repeats
D‐gal: D‐galactose
DRP1: dynamin‐related protein 1
HCs: hair cells
HEI‐OC1: House Ear Institute Organ of Corti 1
L‐OPA1: the long variant of optic atrophy protein 1
MFN1/2: mitochondrial fusion protein 1/2
mtROS: mitochondrial reactive oxygen species
OMA1: metalloendopeptidase OMA1
OPA1: optic atrophy protein 1
PTM: post‐translational modification
SA‐β‐gal: senescence‐associated β‐galactosidase
SGNs: spiral ganglion neurons
SIRT3: sirtuin 3
SOD_2_
: superoxide dismutase 2
S‐OPA1: the short variant of optic atrophy protein 1
WT: wild type
YME1L1: ATP‐dependent zinc metalloprotease YME1L1
ΔΨm: mitochondrial membrane potential

## 1 INTRODUCTION

Age‐related hearing loss (ARHL), also known as presbycusis, is characterized by bilateral, progressive sensorineural hearing loss that develops gradually with age. According to the World Health Organization, at least 65% of individuals over the age of 60 experience hearing loss, and the prevalence of hearing loss increases exponentially with age (World Health Organization, 2021). ARHL poses a significant socio‐economic burden and represents an individual risk factor for both psychological and physical diseases due to the aging of the global population (Jafari et al., 2019; Slade et al., 2020). Current research indicates that the primary pathology affecting the peripheral auditory system in ARHL involves the degeneration and loss of hair cells (HCs) and spiral ganglion neurons (SGNs) (Bermúdez‐Muñoz et al., 2020). The occurrence and development of ARHL are predominantly associated with factors such as mitochondrial dysfunction, oxidative stress, inflammation, abnormal autophagy, and DNA damage (Bowl & Dawson, 2019). However, comprehensive investigations into alterations in mitochondrial dynamics and their impact on ARHL have been limited. Mitochondrial dynamics is a critical pathway for maintaining mitochondrial quality control (Ni et al., 2015). Physiologically, mitochondria maintain the balance of both quantity and quality through fusion and fission processes, ensuring the preservation of normal physiological functions. The equilibrium of this process is known as mitochondrial dynamics homeostasis (Chan, 2020; Yoo & Jung, 2018). Among these processes, mitochondrial fission is primarily mediated by dynamin‐related protein 1 (DRP1) and regulated by fission proteins such as mitochondrial dynamics protein of 49 and 51 and mitochondrial fission factor. In contrast, outer membrane fusion is mainly governed by the mitochondrial fusion proteins MFN1/2, while inner membrane fusion is regulated by optic atrophy protein (OPA1), which also preserves the structural integrity of mitochondrial cristae (Nyenhuis et al., 2023; Sharma et al., 2019). Mutations in OPA1, currently recognized as the sole fusion protein GTPase located in the inner mitochondrial membrane, can lead to syndromic hearing loss (Payne et al., 2004; Santarelli et al., 2015). It is currently understood that the function of OPA1 is primarily regulated through two mechanisms: quantity and activity (Adaniya et al., 2019).

The quantitative regulation of OPA1 is achieved through the proteolytic processing of the long chain (L‐OPA1), which can be cleaved into the short chain (S‐OPA1) by the mitochondrial proteases OMA1 and YME1L1 at the S1 and S2 sites, respectively (Ishihara et al., 2006). This process is a crucial regulatory step in balancing mitochondrial fusion and fission (MacVicar & Langer, 2016; von der Malsburg et al., 2023; Wai et al., 2015). A growing body of evidence suggests that L‐OPA1 is anchored to the inner mitochondrial membrane, where it plays a key role in promoting inner membrane fusion. Conversely, the accumulation of S‐OPA1 unbound to the inner membrane can accelerate mitochondrial fission (Wai et al., 2015), subsequently leading to mitochondrial dysfunction (Lai et al., 2020). In addition to quantity, the function of OPA1 is also subject to regulation through deacetylation modifications (Samant et al., 2014). SIRT3, localized within the mitochondria, serves as the principal deacetylase responsible for mitochondrial protein deacetylation (Wang et al., 2021; Zhang et al., 2020, 2021). Multiple studies have demonstrated that SIRT3 plays a crucial role in the occurrence and development of age‐related neurodegenerative diseases, with its anti‐aging effects primarily achieved through its deacetylase activity (Ji et al., 2022; Zhou et al., 2022). OPA1 is a significant target of SIRT3 (Benigni et al., 2019; Wang et al., 2023). The C‐terminal K926 and K931 lysine sites in the GTPase effector domain of OPA1 are hyperacetylated when SIRT3 is absent, in turn reducing its GTPase activity and leading to dysregulated mitochondrial dynamics (Samant et al., 2014). SIRT3 is reportedly a SUMOylated protein, and SUMOylation inhibits its deacetylase activity, which can be activated by mutating the 223rd SUMOylation site of SIRT3 in mice using CRISPR/Cas9 technology (He, Shangguan, et al., 2021; Wang et al., 2019).

To date, no studies have explored the association between OPA1 and ARHL. Consequently, the contribution of OPA1 to the occurrence and development of ARHL and the underlying mechanisms remain unknown. In this study, we observed a disturbance in mitochondrial dynamics within senescent hair cells. Subsequent investigations revealed a decrease in L‐OPA1 mediated by the activation of the OMA1/L‐OPA1 pathway and an increase in the acetylation level of OPA1 mediated by the inhibition of the SIRT3/OPA1 signaling in senescent hair cells. Furthermore, during the aging process in C57BL/6J mice harboring mutations in the SUMOylation site of SIRT3, we observed that activating the SIRT3/OPA1 signaling could alleviate the disturbance in mitochondrial dynamics and ameliorate the senescence of cochlear hair cells, ultimately mitigating hearing loss in mice. This study underscores the pivotal roles of both the molecular quantity and activity of OPA1 in the occurrence and development of ARHL.

## 2 MATERIALS AND METHODS

### 2.1 Cell culture and reagents

HEI‐OC1 cells were cultured in high glucose Dulbecco's modified Eagle's medium (DMEM; Gibco, C12430500BT) containing 10% fetal bovine serum (FBS; Gibco, 10099141C) and 1% penicillin (Sangon Biotech, B540729) in a 33°C incubator with a CO_2_ concentration of 10%.

D‐galactose (D‐gal; Sangon Biotech, A600215) was dissolved in complete medium and filtered to achieve concentrations of 20, 40, and 60 mg/mL. The HEI‐OC1 cells were treated with these concentrations for 24 h to establish the senescence model. MYLS22 (MedChemExpresss, HY‐136446), Tyrphostin A9 (MedChemExpresss, HY‐15511), and kaempferol (MedChemExpresss, HY‐14590) were dissolved in dimethyl sulfoxide (DMSO) and diluted to final concentrations with complete medium. The MYLS22 group was pretreated with 10 μM for 6 h, followed by treatment with D‐gal (40 mg/mL) for 24 h. The Tyrphostin A9 group was pretreated with 5 μM for 6 h, followed by treatment with D‐gal (40 mg/mL) for 24 h. The kaempferol group was pretreated with 5 μM for 6 h and then co‐treated with D‐gal (40 mg/mL) for 24 h.

### 2.2 Animals

Experimental C57BL/6J mice were divided into four groups: 6‐month‐old wild‐type (WT) and K223R groups, as well as 24‐month‐old WT and K223R groups. These mice were generously provided by the laboratory of Professor Jinke Cheng (School of Basic Medicine, Shanghai Jiao Tong University). SIRT3 K223R mice were constructed using CRISPR/Cas9 technology (He, Shangguan, et al., 2021; Wang et al., 2019). None of the animals had any history of ototoxic injury or noise exposure. All animals were housed and bred in a standard SPF‐grade experimental environment at the Experimental Animal Center of Shanghai Jiao Tong University School of Basic Medicine. The use of animals in the experiments was approved by the Experimental Animal Ethics Committee of Shanghai Jiao Tong University School of Medicine.

### 2.3 Cell viability

Cell Counting Kit‐8 (CCK‐8; Dojindo, CK04) was used to assess cell viability in accordance with the instructions provided. HEI‐OC1 cells were cultured in 96‐well plates at a density of 5 × 10^4^ cells/mL and incubated in six replicate wells overnight. After drug treatment, cells were incubated with the CCK‐8 reagent for 2 h. Then, optical density values were measured at 450 nm using a microplate reader (Tecan, Switzerland).

### 2.4 Flow cytometry

DCFH‐DA (Yeasen, 50101ES01) was used to detect total cellular ROS, and mitoROS™ 580 (AAT Bioquest, AAT‐B16052) was used to detect intracellular mtROS. Cells were incubated with probes in the dark as instructed, washed with PBS, and then collected with trypsin. HEI‐OC1 cells were collected, centrifuged, and prepared as single‐cell suspensions in PBS. Finally, flow cytometric analyses were performed (Beckman Coulter, USA). ROS levels were detected using the FITC channel, while mtROS levels were detected using the PE channel.

### 2.5 ATP content

ATP content was detected using an ATP kit (Beyotime, S0026) in accordance with the instructions provided. Cells were lysed and centrifuged at 12,000*g* for 5 min at 4°C. The supernatant was collected for subsequent analyses, with three replicate wells per sample. Then, 100 μL of working solution was added to each well, and the plates were incubated for 5 min while protected from light. Following the addition of the sample or standard to be tested, chemiluminescence detection was performed using a multifunctional microplate reader (Tecan, Switzerland). ATP content was calculated based on standard curves and protein quantification.

### 2.6 Mitochondrial membrane potential (ΔΨm) detection

ΔΨm detection was performed using the JC‐1 probe (Beyotime, C2006) in accordance with the instructions provided. This probe exists as a green‐fluorescent monomer at lower ΔΨm conditions and can form red‐fluorescent aggregates in the mitochondrial matrix under higher ΔΨm conditions.

### 2.7 Immunoprecipitation

Protein immunoprecipitation was performed using a Protein A/G immunoprecipitation kit (Biolinkedin, IK‐1004) in accordance with the instructions provided. Immune complexes were prepared from protein samples using anti‐acetylated lysine (CST, 9441S, 1:100), anti‐SIRT3 (CST, 5490S, 1:100), or control anti‐IgG (Proteintech, 30000‐0‐AP, 1:100). These complexes were bound to Protein A/G magnetic beads and subsequently collected. Western blotting was conducted using anti‐OPA1 (BD Biosciences, 612606, 1:1000), and band intensity was quantified.

### 2.8 Western blotting

HEI‐OC1 cells were lysed with pre‐chilled RIPA lysis buffer (Epizyme, PC101) containing protease inhibitor (Epizyme, GRF101) and phosphatase inhibitor (Epizyme, GRF102). Equal amounts of protein extracts were electrophoresed on 10% or 12.5% SDS‐PAGE gels and subsequently transferred to polyvinylidene difluoride membranes. Following blocking with 5% skim milk at room temperature for 2 h, the blots were incubated with the following primary antibodies at 4°C overnight: anti‐OPA1 (BD Biosciences, 612606, 1:1000), anti‐DRP1 (CST, 8570S, 1:1000), anti‐MFN1 (Beyotime, AF7461, 1:1000), anti‐MFN2 (Proteintech, 12186‐1‐AP, 1:1000), anti‐OMA1 (Santa Cruz, sc‐515788, 1:800), anti‐YME1L1 (Proteintech, 11510‐1‐AP, 1:1000), anti‐SIRT3 (CST, 5490S, 1:1000), anti‐Ac‐SOD_2_ (acetyl K68) (Abcam, ab137037, 1:1000), anti‐NALP1 (CST, 4990S, 1:1000), anti‐NLRP3 (Servicebio, GB114320, 1:1000), anti‐caspase 1 (Proteintech, 22915‐1‐AP, 1:2000), anti‐IL‐1β (Absin, abs120224, 1:2000), anti‐IL‐18 (Proteintech, 10663‐1‐AP, 1:3000), anti‐TNFα (Proteintech, 17590‐1‐AP, 1:1000), anti‐P53 (Proteintech, 10442‐1‐AP, 1:1000), anti‐phospho‐P53 (Proteintech, 28961‐1‐AP, 1:1000), and anti‐P21 (Proteintech, 28248‐1‐AP, 1:1000). Reference proteins were detected using anti‐GAPDH (Proteintech, 60004‐1‐Ig, 1:3000), anti‐β actin (Proteintech, 66009‐1‐Ig, 1:3000), and anti‐α‐Tubulin (Proteintech, 11224‐1‐AP, 1:3000). HRP‐labeled goat anti‐mouse IgG (Beyotime, A0216, 1:3000) and anti‐rabbit IgG (Beyotime, A0208, 1:3000) were employed as secondary antibodies. An ECL kit (Epizyme, SQ202L) was utilized for protein band detection. ImageJ was employed for the quantification of band intensities, which were subsequently normalized to the intensity values of the reference protein.

### 2.9 SA‐β‐gal senescence staining

SA‐β‐gal staining was performed using the cellular senescence β‐galactosidase staining kit (Beyotime, C0602) in accordance with the instructions provided. After washing the cells with PBS and subsequent fixation, staining solution was added, followed by sealing with parafilm and overnight incubation at 37°C. Then, the cell climbing slices were removed and imaged with an upright optical microscope.

### 2.10 Mitochondrial morphological analyses

Mitochondrial morphology staining of HEI‐OC1 cells was conducted using the mitotracker red probe (100 nM; Yeasen, 40741ES50). Following incubation at 33°C for 30 min, the specimens were observed by laser confocal microscopy.

### 2.11 Auditory brainstem response (ABR)

The detection of mouse ABR waveforms was conducted using the Tucker Davis Technologies (TDT) system III audiology testing workstation. All mice were anesthetized by intraperitoneal injection (ketamine 100 mg/kg, xylazine 10 mg/kg). Subcutaneous electrodes were inserted at the top of the median skull, the otomastoid portion, and the contralateral thigh. ABR waveforms were recorded at sound pressure levels (SPLs). The recordings were conducted using the BioSigRZ software after calibration. The hearing threshold was established as the minimum sound intensity that produced a reproducibly sustained ABR waveform.

### 2.12 Cochlear histological staining

The dissected cochleae were perfused with 4% paraformaldehyde (PFA) (Servicebio, China) and fixed overnight. The cochleae were then immersed in 10% EDTA decalcification solution (Servicebio, China) for 1 week at room temperature for decalcification, followed by dehydration with an ethanol gradient and paraffin embedding. Paraffin‐embedded sections were prepared from the middle part of the cochleae using a pathology microtome (Leica, Germany). Cochlear sections containing at least four organs of Corti were selected for subsequent staining. The cochlear paraffin‐embedded sections were dewaxed, hydrated, and stained with hematoxylin and eosin (H&E) (Beyotime, C0105). Images were captured following dehydration and mounting.

### 2.13 Cochlear immunohistochemistry (IHC) staining

The prepared cochlear paraffin‐embedded sections were dewaxed, hydrated, and antigenically repaired, followed by blocking with 3% hydrogen peroxide and 3% bovine serum albumin (BSA) at room temperature for 30 min. The samples were then incubated with primary anti‐OPA1 (Abcam, ab157457), anti‐Ac‐SOD_2_ (acetyl K68) (Abcam, ab137037), or acetylated‐lysine (CST, 9441S) at 4°C overnight, followed by incubation with an HRP‐labeled goat anti‐rabbit secondary antibody (Beyotime, A0208) at room temperature for 1 h. Staining with 3,3‐diaminobenzidine and counterstaining with hematoxylin were then conducted, followed by dehydration, mounting, and image collection. ImageJ was used to perform semi‐quantitative analyses of the staining results.

### 2.14 Immunofluorescent staining

The apical, middle, and basal turns of the cochlear basilar membrane were dissected using a stereoscopic dissecting microscope (Zeiss). The samples were treated with 0.2% Triton X‐100 (Sigma, 9002‐93‐1) and 10% BSA (Beyotime, ST025) at room temperature for 30 min, followed by incubation with anti‐Prestin (Santa Cruz, sc‐22692) and anti‐vGlut3 (Synaptic Systems, 135203) primary antibodies at 4°C overnight. Then, the samples were incubated with fluorescent secondary antibodies (Invitrogen, A32814, A21207) for 1 h at room temperature, and Hoechst (Beyotime, C1011) was used for nuclear staining. Hair cells in the three turns were observed and counted using laser confocal microscopy. Prestin (green fluorescence) specifically stains outer hair cells, while vGlut3 (red fluorescence) specifically stains inner hair cells, and Hoechst (blue fluorescence) stains the nuclei.

When performing the immunofluorescent staining of HEI‐OC1 cells, the cells were fixed with 4% PFA (Servicebio, China) at room temperature for 15 min, followed by permeabilization and blocking with immunostaining blocking solution (Beyotime, P0260) containing Triton X‐100 for 20 min. Subsequently, the cells were incubated with primary anti‐SIRT3 (Proteintech, 10099‐1‐AP) overnight at 4°C. The cells were then incubated with Alexa Fluor 488 donkey anti‐rabbit IgG (Yeasen, 34206ES60) for 1 h at room temperature, and DAPI (Beyotime, P0131) was used for nuclear staining. Subsequently, images were collected using an upright fluorescence microscope.

### 2.15 Transmission electron microscopy (TEM)

The cochleae were fixed in 2.5% glutaraldehyde fixative (Servicebio, G1102) overnight, decalcified using 10% EDTA decalcification solution (Servicebio, G1105) at room temperature for 1 week, and fixed in 1% osmic acid (Sigma‐Aldrich, O5500) at room temperature for 2 h. After gradient dehydration, the cochleae were embedded in epoxy resin parallel to the cochlear axis. Semi‐thin sections with a thickness of 2.0 μm were taken, and 1% toluidine blue staining was performed to detect the intact cochlear axis. Ultrathin sections were prepared using an ultramicrotome (Leica, UC7). These sections were double‐stained with 3% uranyl acetate and lead citrate for 15 min. A transmission electron microscope (JEM‐1200EX, Japan) was then used to observe the morphology and mitochondrial ultrastructural characteristics of these cochlear hair cells.

### 2.16 Statistical analysis

Statistical analyses were performed using GraphPad Prism 8.0 (GraphPad Software, Inc., San Diego, CA, USA). All data are expressed as the mean ± *SD*. All experiments were repeated at least three times (*n* ≥ 3). The homogeneity of variances in the data was assessed using Levene's test. Comparisons between two groups were performed using two‐tailed unpaired *t*‐tests, whereas comparisons between more than two groups employed one‐way ANOVA. The Brown‐Forsythe and Welch ANOVA tests and Dunnett T3 tests were used in cases of uneven variance. Two‐way ANOVA was used when assessing comparisons between groups in ABR analyses. *p* < 0.05 was considered statistically significant.

## 3 RESULTS

### 3.1 Senescent hair cells exhibit mitochondrial dysfunction

We treated HEI‐OC1 cells with different concentrations of D‐galactose (D‐gal) for 6, 24, or 48 h and then analyzed the cell viability (Figure S1a). Ultimately, an *in vitro* model of gradient senescence in cochlear hair cells was established via a 24‐h treatment with D‐gal at concentrations of 20, 40, and 60 mg/mL. SA‐β‐gal staining (Figure S1b,c) and the detection of senescence marker proteins including P53, phosphorylated P53 (p‐P53), and P21 (Figure S1d,e) indicated that the senescence of hair cells worsened as the concentration of D‐gal increased. Additionally, senescent hair cells exhibited decreased ATP content and increased intracellular ROS levels (Figure S1f–h). To specifically assess the contribution of the mitochondrial respiratory chain to intracellular ROS production and eliminate the influence of other potential sources (Orrenius et al., 2007; Sinha et al., 2013), we quantified the content of mitochondrial ROS (mtROS), revealing progressive increases over the course of senescence (Figure S1i,j). Our findings indicated that the increased intracellular ROS production was primarily attributed to mitochondrial functional impairment, confirming the existence of mitochondrial dysfunction during hair cell senescence.

### 3.2 Senescent hair cells exhibit excessive mitochondrial fission and OPA1 dysfunction

**FIGURE 1:**
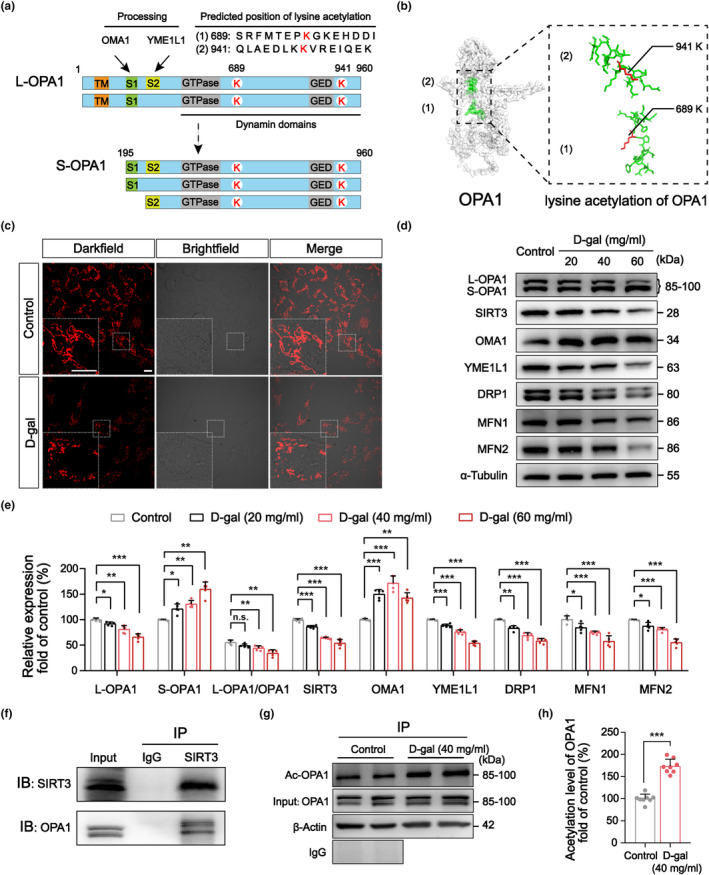
Senescent HEI‐OC1 cells exhibit dysregulated mitochondrial dynamics and OPA1 abnormalities. (a) Protein structures of L‐OPA1 and S‐OPA1. (b) Three‐dimensional structure of human OPA1 and its two lysine acetylation sites. (c) Mitotracker staining revealed that mitochondrial morphology exhibited a tendency toward fragmentation in senescent HEI‐OC1 cells after D‐gal (40 mg/mL) treatment for 24 h. Scale bar: 5 μm. (d, e) Alterations in the expression of mitochondrial dynamics‐related proteins by western blotting suggested the dysregulation of mitochondrial dynamics. *n* = 5. (f) The direct interaction between SIRT3 and OPA1 in senescent HEI‐OC1 cells was discerned through immunoprecipitation. (g, h) Statistical analyses of acetylated OPA1 levels as detected through immunoprecipitation experiments confirmed the increased acetylation of OPA1 in senescent hair cells. *n* = 8. *p < 0.05, **p < 0.01, and ***p < 0.001 versus the control group; n.s. no statistical difference; one‐way ANOVA.

Mitochondrial dynamics are pivotal for the maintenance of mitochondrial function (Chan, 2020; Chen et al., 2023; Quintana‐Cabrera & Scorrano, 2023). We next predicted the lysine acetylation sites present in the human, mouse, and rat OPA1 proteins and identified two identical, conserved acetylation sites in all three species, located at 689K and 941K in dynamin domains (Figure 1a,b). This discovery suggests that post‐translational modification (PTM) of OPA1 may play a substantial role in its molecular function.

To assess whether mitochondrial dynamics and morphology are altered in senescent hair cells, we examined mitochondrial morphology and analyzed the expression of dynamics‐related proteins in HEI‐OC1 cells. Mitochondrial tracing revealed excessive mitochondrial fragmentation following treatment with D‐gal (40 mg/mL) for 24 h, with a gradual shift from elongated forms to fragmented punctate structures (Figure 1c). Western blotting for dynamics‐related proteins indicated a decrease in the proportion of L‐OPA1 and a corresponding increase in the levels of S‐OPA1. Furthermore, the levels of MFN1/2 and DRP1 were decreased in senescent cells after D‐gal treatment. We also found a significant increase in the levels of hydrolase OMA1, which was consistent with the alterations in OPA1. In contrast, the expression of another hydrolase, YME1L1, decreased (Figure 1d,e). Our findings indicate that excessive cleavage of L‐OPA1 likely induced by OMA1 activation occurs in senescent hair cells, inhibiting mitochondrial inner membrane fusion and leading to mitochondrial dysfunction.

Furthermore, we found a reduction in the deacetylase SIRT3 in senescent hair cells (Figure 1d,e) and identified the direct interaction between SIRT3 and OPA1 through immunoprecipitation (Figure 1f). Subsequently, we also found that the acetylation level of OPA1 in senescent hair cells was significantly increased (Figure 1g,h), suggesting that it may be related to the reduction in SIRT3. These results suggest that in addition to excessive cleavage of L‐OPA1, the enhanced acetylation of OPA1 may also contribute to the emergence of mitochondrial dynamics disorder and mitochondrial dysfunction in hair cells.

### 3.3 L‐OPA1 inhibition exacerbates the dysregulation of mitochondrial dynamics and hair cell senescence

To investigate the relationship between dysregulated mitochondrial dynamics mediated by L‐OPA1 and hair cell senescence, we employed two small‐molecule compounds, namely a specific OPA1 inhibitor (MYLS22) (Corrado et al., 2021; Herkenne et al., 2020; Noguchi et al., 2023) and a mitochondrial fission inducer (Tyrphostin A9) (Park et al., 2011). Our aim was to inhibit mitochondrial fusion and observe the effects of L‐OPA1 alterations on hair cell senescence. Our results revealed that both compounds reduced the viability of senescent hair cells (Figure S2a). We thus selected 10 μM MYLS22 and 5 μM Tyrphostin A9 doses for subsequent experiments in senescent hair cells induced by D‐gal. Western blotting demonstrated that the expression of L‐OPA1 in senescent hair cells was further significantly reduced after treatment with MYLS22 or Tyrphostin A9. MYLS22 exhibited inhibitory effects on both L‐OPA1 and S‐OPA1, whereas Tyrphostin A9 specifically inhibited L‐OPA1. Notably, there were no significant changes in other dynamics‐related proteins, such as DRP1 or MFN1 (Figure S2b,c). Furthermore, Mitotracker staining revealed an increase in fragmented mitochondria and further disruption of dynamic homeostasis following L‐OPA1 inhibition (Figure S2d).

**FIGURE 2:**
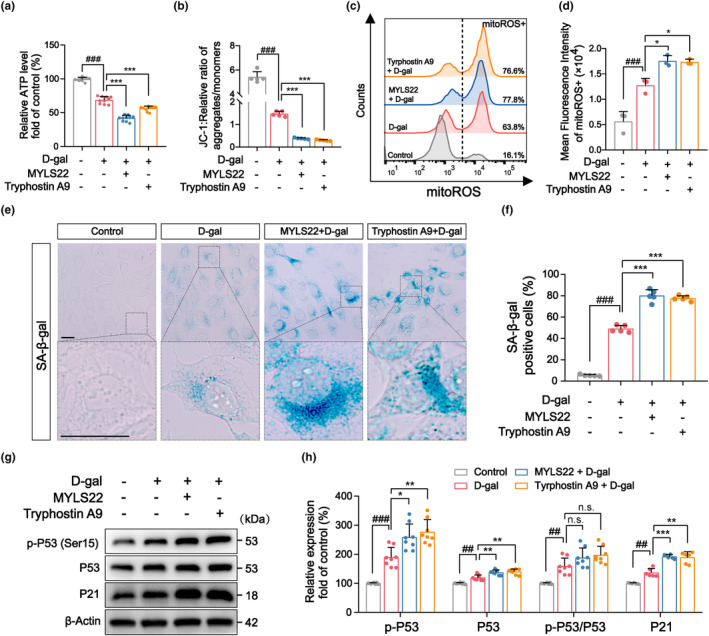
L‐OPA1 inhibition aggravates mitochondrial dysfunction and cellular senescence in HEI‐OC1 cells. (a) Analyses of ATP content revealed that the addition of MYLS22 or Tyrphostin A9 reduced ATP production in senescent cells. *n* = 10. (b) The detection of mitochondrial membrane potential (ΔΨm) levels indicated that the ΔΨm was reduced after L‐OPA1 inhibition. *n* = 5. (c, d) MtROS content and mean fluorescence intensity (MFI) levels in senescent HEI‐OC1 cells were measured by flow cytometry in the PE channel, showing that mtROS accumulation increased significantly after L‐OPA1 inhibition. *n* = 3. (e, f) SA‐β‐gal staining revealed that L‐OPA1 inhibition aggravated hair cell senescence and elevated the proportion of senescent cells. Scale bar: 10 μm. *n* = 5. (g, h) Western blotting results focusing on the expression of senescence marker proteins in senescent HEI‐OC1 cells suggested that p‐P53 and P21 levels were increased significantly after L‐OPA1 inhibition. *n* = 6. ^##^ *p* < 0.01 and ^###^ *p* < 0.001 versus the control group; *p < 0.05, **p < 0.01, and ***p < 0.001 versus the D‐gal group; n.s. no statistical difference; one‐way ANOVA.

Evaluation of mitochondrial function indicated further mitochondrial dysfunction in senescent hair cells after MYLS22 or Tyrphostin A9 treatment, including a further decrease in ATP content and mitochondrial membrane potential (ΔΨm), as well as a further increase in mtROS levels (Figure 2a–d). These results suggest that reducing the absolute quantity of L‐OPA1 can directly impair the mitochondrial function of senescent hair cells. Subsequently, the analysis of aging markers confirmed that L‐OPA1 inhibition exacerbated hair cell senescence, as evidenced by an increased percentage of SA‐β‐gal senescence staining‐positive cells (Figure 2e,f) and the upregulation of senescence marker proteins including p‐P53, P53, and P21 (Figure 2g,h). These findings indicate that the inhibition of L‐OPA1 can worsen the dysregulation of mitochondrial dynamics and lead to the senescence of cochlear hair cells.

### 3.4 SIRT3‐mediated OPA1 deacetylation alleviates cochlear hair cell senescence

In addition to the alteration of long‐ and short‐chain forms, the activity of OPA1 is also regulated by SIRT3‐mediated deacetylation (Adaniya et al., 2019). In this study, in addition to elevated levels of OPA1 acetylation, a decrease in SIRT3 expression was also observed (Figure 1d,e). Therefore, we speculate that increased levels of OPA1 deacetylation may play a protective role in the aging process of cochlear hair cells. Here, we substantiated this hypothesis through activating SIRT3 with kaempferol (Li et al., 2022; Marfe et al., 2009), a small polyphenolic molecule widely found in plants, which has been shown in several studies to ameliorate a variety of neurodegenerative diseases including Alzheimer's disease (Holland et al., 2020; Xie et al., 2022) and cognitive impairment (Li et al., 2022).

**FIGURE 3:**
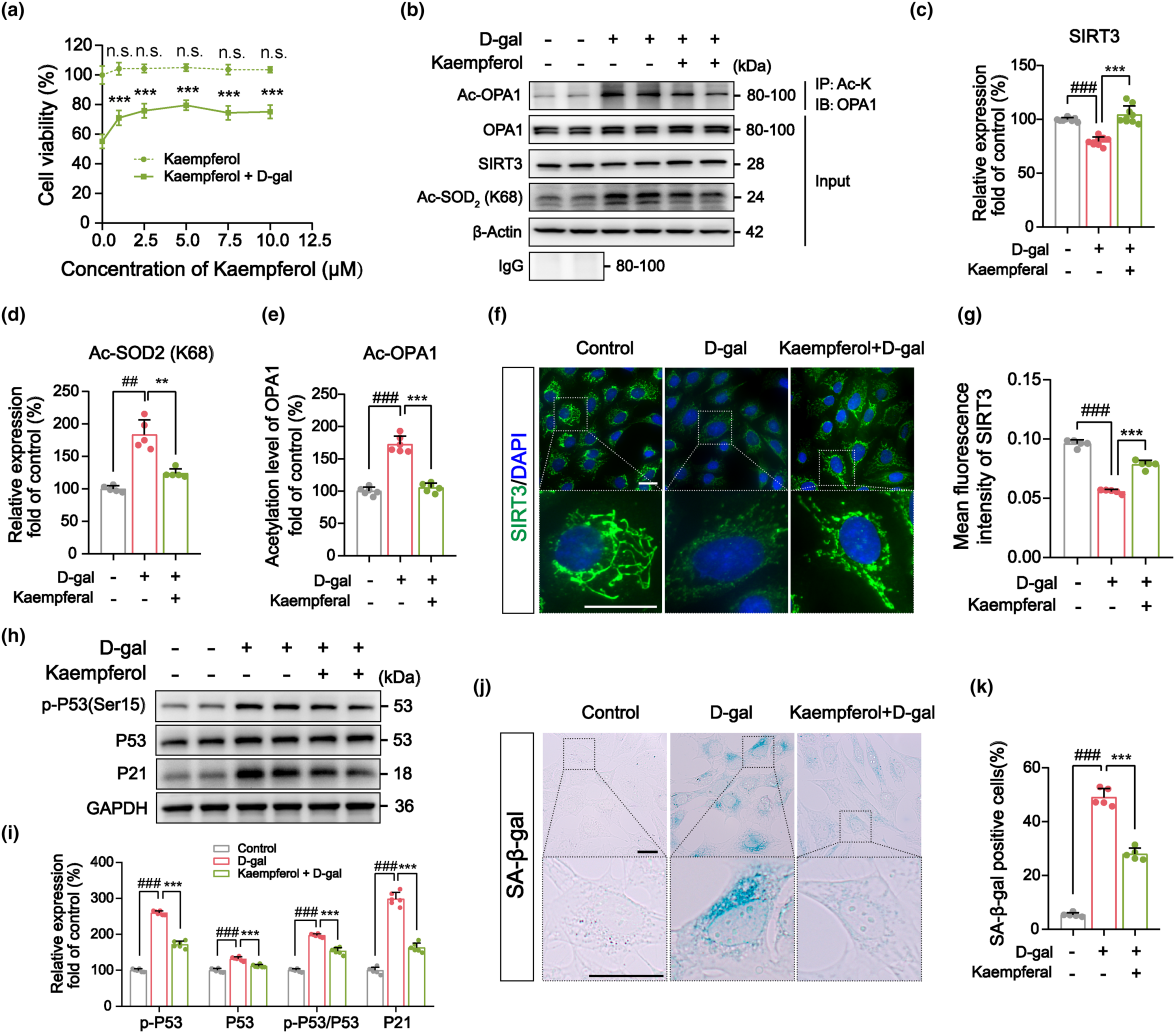
Reducing OPA1 acetylation levels via the kaempferol‐mediated activation of SIRT3 ameliorates aberrant mitochondrial dynamics and the senescence of cochlear hair cells. (a) CCK‐8 assays revealed that kaempferol ameliorated the cytotoxic effects of D‐gal treatment on HEI‐OC1 cells (40 mg/mL, 24 h). (b–e) Immunoprecipitation and western blotting showed that kaempferol enhanced SIRT3 function, including elevated SIRT3 expression (*n* = 9) and reduced acetylated SOD_2_ (*n* = 5)_,_ as well as reduced acetylated OPA1 levels (*n* = 6) in senescent HEI‐OC1 cells. (f, g) Immunofluorescent staining for SIRT3 and analyses of mean fluorescence intensity (MFI) similarly revealed that kaempferol enhanced SIRT3 expression and improved mitochondrial morphology. Green: SIRT3 staining, located to the mitochondria. Blue: DAPI staining, located to the nucleus. Scale bar: 10 μm. *n* = 5. (h, i) Statistical analyses of western blotting results pertaining to the expression of senescence marker proteins in senescent HEI‐OC1 cells suggested that p‐P53 and P21 levels significantly decreased after the activation of the SIRT3/OPA1 signaling by kaempferol. *n* = 6. (j, k) SA‐β‐gal staining and the proportion of senescence‐positive HEI‐OC1 cells indicated that the senescence phenotype was alleviated after kaempferol treatment. Scale bar: 10 μm. *n* = 5. ^##^ *p*  < 0.01 and ^###^ *p* < 0.001 versus the control group; **p  < 0.01 and ***p < 0.001 versus the D‐gal group; n.s. no statistical difference; one‐way ANOVA.

We found that the treatment of senescent HEI‐OC1 cells with a small dose of kaempferol significantly improved cell viability (Figure 3a). Subsequently, we selected 5 μM kaempferol as a dose for use in further experiments. We discovered that the addition of kaempferol in senescent HEI‐OC1 cells increased SIRT3 expression and decreased the level of acetylated SOD_2_ (Ac‐SOD_2_), the main target for SIRT3, indicating that SIRT3 function was improved (Figure 3b–d). In addition, the levels of OPA1 acetylation were significantly reduced after kaempferol treatment as detected via immunoprecipitation (Figure 3b,e). Immunofluorescence staining corroborated these findings, demonstrating a significant improvement in mitochondrial morphology in the kaempferol + D‐gal group (Figure 3f,g). The results of aging marker detection and SA‐β‐gal staining revealed that kaempferol diminished the expression of p‐P53/P53 and P21 and reduced the proportion of senescence‐positive HEI‐OC1 cells (Figure 3h–k). These data suggested that reducing OPA1 acetylation levels via the activation of SIRT3 was sufficient to remediate dysregulated mitochondrial dynamics and alleviate the senescence of cochlear hair cells.

### 3.5 SIRT3 activation‐associated reductions in OPA1 acetylation attenuate mitochondrial structural damage in the cochlear hair cells of ARHL mice

**FIGURE 4:**
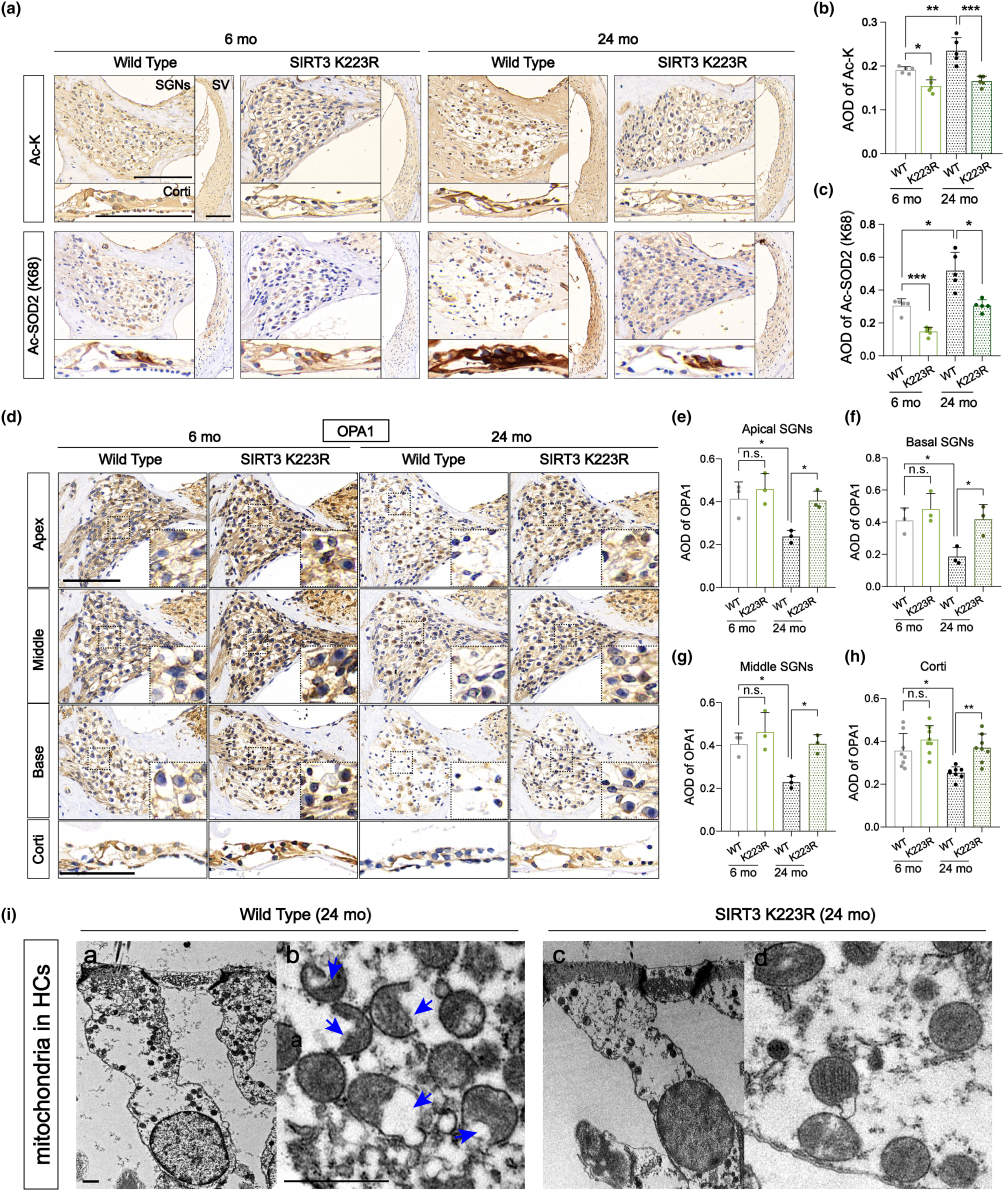
Reducing levels of OPA1 acetylation by activating SIRT3 attenuates mitochondrial structural damage in the cochlear hair cells of ARHL mice. (a–c) Immunohistochemistry (IHC) staining of acetylated SOD_2_ and acetylated lysine revealed that the average optical density (AOD) value and positive staining area in the K223R group were significantly reduced relative to those in the WT group. Scale bar: 100 μm. *n* = 5. (d–h) OPA1 staining and AOD analyses revealed that OPA1 expression in the cochlea of 24‐month‐old mice in the K223R group exhibited a pronounced increase as compared to the WT group. Scale bar: 100 μm. *n* = 3 per turn and *n* = 8 per Corti. (i) TEM was utilized to observe mitochondrial structures in cochlear hair cells of 24‐month‐old SIRT3 deSUMOylated and WT mice. In the WT group, discernible alterations were evident in the mitochondria of cochlear hair cells, which exhibited significant swelling, disrupted or absent cristae structures, and the presence of vacuolization (indicated by blue arrows). Conversely, the mitochondria of hair cells in the SIRT3 K223R group exhibited a preserved, normal morphology. Scale bar: 1 μm. *p < 0.05, **p < 0.01, and ***p < 0.001 vs. the WT group; n.s. no statistical difference; one‐way ANOVA.

Given our *in vitro* results indicating the pivotal involvement of SIRT3/OPA1 signaling in the aging process of cochlear hair cells, we devised an *in vivo* investigation to examine the impact of SIRT3 activation and the subsequent reduction of OPA1 acetylation levels on the mitochondria of cochlear hair cells in aging mice. Both 6‐ and 24‐month‐old C57BL/6J mice with deSUMOylated SIRT3 (SIRT3 K223R) were employed for these studies. We conducted Ac‐SOD_2_ and acetylated lysine (Ac‐K) staining in the cochlea of the mice, revealing that acetylation levels in the 24‐month‐old WT mice exceeded those in their 6‐month‐old counterparts, especially noticeable in the Ac‐SOD_2_ levels in hair cells. In comparison with WT mice, cochlear acetylation levels were significantly reduced in both 6‐month‐old and 24‐month‐old SIRT3 deSUMOylated mice (Figure 4a–c). Additionally, we analyzed OPA1 expression, revealing a pronounced age‐related decline in OPA1 expression within the cochlear organ of Corti and the SGNs of apical, middle, and basal turns in the 24‐month‐old WT group compared with the younger group. In contrast, the cochlear organ of Corti and SGNs in 24‐month‐old SIRT3 deSUMOylated mice exhibited significantly higher OPA1 expression compared with that in age‐matched WT mice (Figure 4d–h).

Subsequently, we proceeded to evaluate the impact of SIRT3/OPA1 signaling on the mitochondria of cochlear hair cells in aged mice. We found a substantial abundance of markedly swollen and fragmented mitochondria in the cochlear hair cells of 24‐month‐old WT mice under TEM. The structural integrity of cristae was disrupted, broken, or even absent. These mitochondria also showed pronounced vacuolation (Figure 4i (a, b)). In contrast, within the cochlear hair cells of age‐matched SIRT3 deSUMOylated mice, mitochondrial morphology remained essentially unaltered, presenting a virtually normal appearance. Furthermore, the mitochondrial cristae structure appeared orderly and distinctly discernible (Figure 4i (c, d)). These findings strongly support the notion that SIRT3 activation to enhance OPA1 function can significantly mitigate the age‐related deterioration in mitochondrial morphology and structural integrity, thereby preserving normal mitochondrial function.

### 3.6 Activation of SIRT3/OPA1 signaling attenuates cochlear senescence and ARHL in mice

**FIGURE 5:**
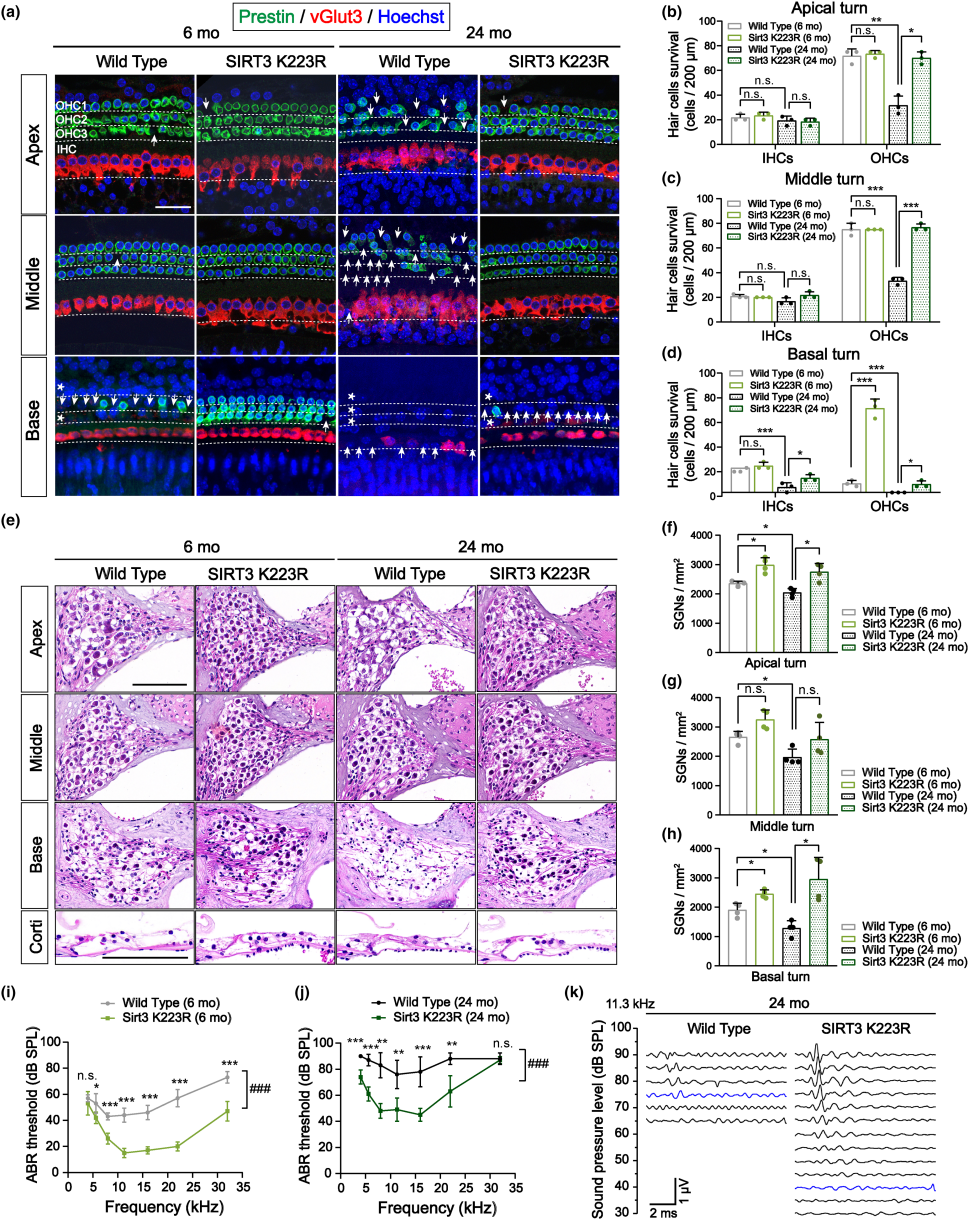
Reducing levels of OPA1 acetylation by activating SIRT3 alleviates the age‐related loss of inner and outer cochlear hair cells and the degeneration of SGNs. (a–d) Immunofluorescent staining results from inner hair cells (IHCs) and outer hair cells (OHCs) in the apical, middle, and basal turns of mouse cochlear basilar membrane. Green: Prestin‐stained OHCs; red: vGlut3‐stained IHCs; blue: Hoechst‐stained nuclei. Scale bar: 40 μm. Arrows indicate the absence of hair cells, and asterisks indicate the absence of entire rows of hair cells. Statistically, the loss of OHCs in basal turns in the 6‐month‐old K223R group as well as IHCs in basal turns and OHCs in all turns in the 24‐month‐old K223R group was significantly reduced as compared to the WT group. *n* = 3. *p < 0.05, **p < 0.01, and ***p < 0.001 vs. the WT group; n.s. no statistical difference; one‐way ANOVA. (e–h) H&E staining results for cochlear SGNs and the organ of Corti from mice in the K223R and WT groups at 6 and 24 months of age. Scale bar: 100 μm. The number of preserved SGNs exhibited varying degrees of improvement in both the 6‐month‐old and 24‐month‐old K223R groups. *n* = 4. *p < 0.05, **p < 0.01, and ***p < 0.001 versus the WT group; n.s. no statistical difference; one‐way ANOVA. (i, j) Average ABR thresholds at frequencies of 4, 5.6, 8, 11.3, 16, 22, and 32 kHz in the K223R group and WT group at 6 months and 24 months of age. *n* = 5. *p < 0.05, **p < 0.01, and ***p < 0.001 vs. the WT group; Two‐tailed unpaired *t*‐test. ^###^ *p* < 0.001 indicates a significant difference between the overall ABR thresholds of the K223R and WT groups; two‐way ANOVA. (k) Example of ABR waveforms recorded at 11.3 kHz in 24‐month‐old mice.

Given our *in vitro* observations demonstrating that the activation of SIRT3 and consequent OPA1 deacetylation were sufficient to significantly attenuate age‐related mitochondrial structural damage in cochlear hair cells, we sought to further investigate whether such SIRT3‐mediated intervention holds the potential to protract cochlear senescence and ameliorate ARHL in vivo. We observed and quantified the numbers of inner and outer hair cells in the cochlea of 6‐month‐old and 24‐month‐old SIRT3 deSUMOylated mice. The results demonstrated that in 6‐month‐old mice, the integrity of both inner and outer hair cells in the apical, middle, and basal turns of the K223R group remained preserved with no discernible loss, while the outer hair cells of the cochlear basal turns showed significant loss in the age‐matched WT group (Figure 5a–d). In the 24‐month‐old mice, there was a severe loss in the outer hair cells of all turns and the inner hair cells of basal turns in the WT group. However, although the K223R group also showed hair cell loss in the aforementioned cochlear turns, the extent of the loss was noticeably milder compared to the corresponding turns in the WT group (Figure 5a–d).

Morphological observation and quantification of cochlear SGNs in apical, middle, and basal turns revealed significantly reduced SGNs in the three turns in the 24‐month‐old WT group relative to the 6‐month‐old WT group. Additionally, the SGNs appeared swollen or consolidated, with the corresponding enlargement of intercellular gaps and vacuolated cytoplasmic degeneration. Furthermore, the K223R group exhibited significantly greater preservation of SGNs as compared to the age‐matched WT group (Figure 5e–h). Additionally, the disappearance of hair cell structures and the occurrence of tunnel disintegration and collapse in the organ of Corti were also noticeably less pronounced in the K223R group (Figure 5e–h). To visually confirm the protective effect of SIRT3/OPA1 signaling on auditory function, we conducted ABR tests and discovered that the hearing thresholds of mice in the 24‐month‐old WT group were significantly higher than those of 6‐month‐old WT mice. Moreover, the ABR thresholds of both the 6‐ and 24‐month‐old K223R groups were significantly lower than those of age‐matched WT mice (Figure 5i–k). Our results indicated that the activation of the SIRT3/OPA1 signaling substantially ameliorated cochlear degeneration and mitigated ARHL in aged mice.

### 3.7 Pyroptosis‐related NLRP1/3‐caspase 1 inflammatory pathway activation mediates cochlear hair cell senescence induced by abnormal L‐OPA1

Pyroptosis is a recently identified form of inflammasome‐dependent programmed cell death that can be activated by mitochondrial dysfunction (Swanson et al., 2019). The classical inflammasome pathway is initiated by the activation of caspase 1, which subsequently triggers various inflammasomes and leads to the release of inflammatory factors including IL‐1β and IL‐18 (Bertheloot et al., 2021; Shi et al., 2015). Recent research has unveiled the activation of this inflammatory pathway in models of ototoxicity‐induced hearing loss (Wu et al., 2022) and ARHL (Yang et al., 2023).

**FIGURE 6:**
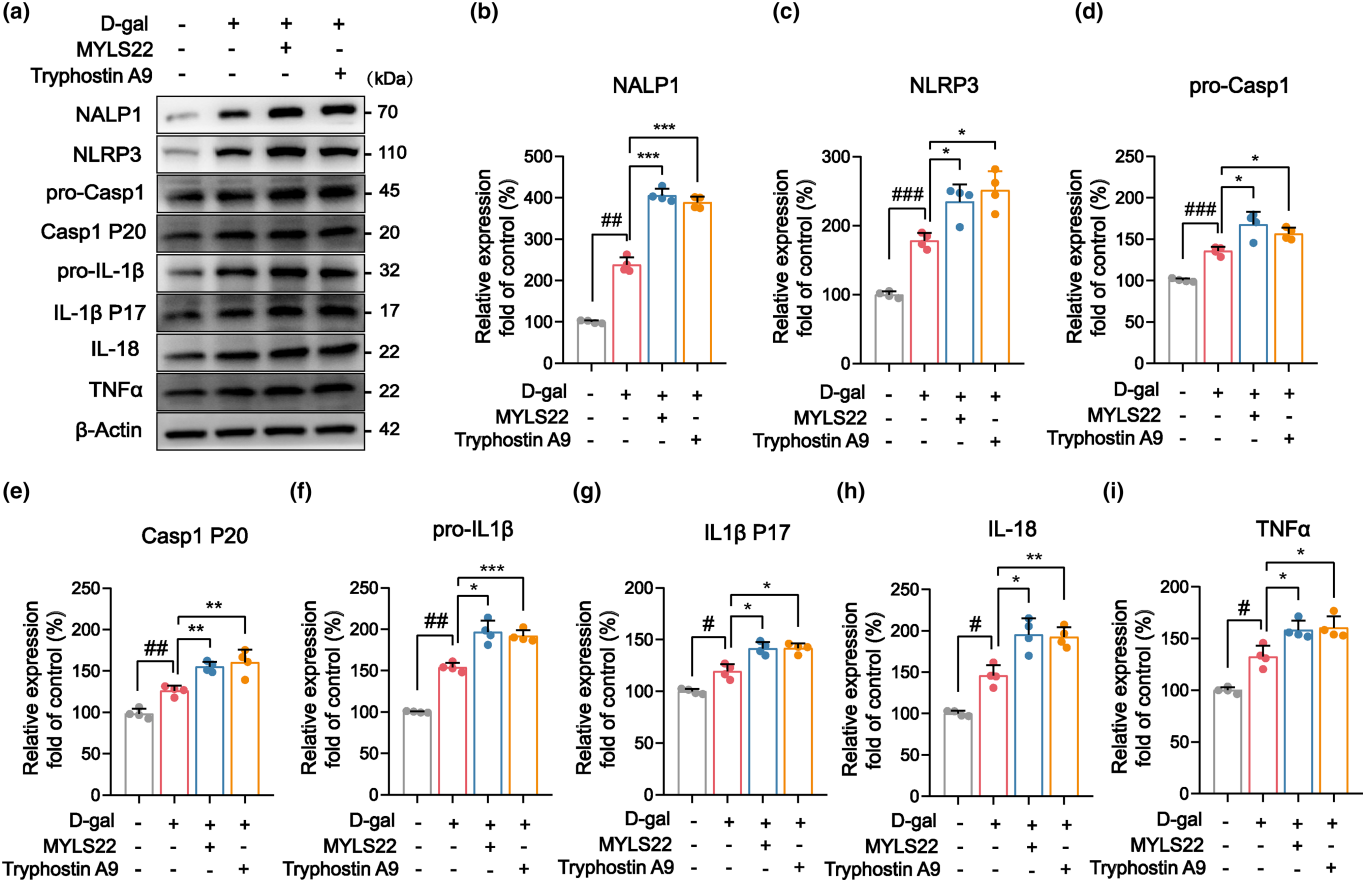
Dysregulated mitochondrial dynamics can activate pyroptosis through the NLRP1/3‐caspase 1 inflammatory pathway. (a) Western blotting revealed changes in the expression of inflammatory pathway molecules in senescent HEI‐OC1 cells. (b–i) Statistical analyses of western blotting results indicated that the inhibition of L‐OPA1 resulted in significant increases in the expression of inflammatory factors, including NLRP1/3, caspase 1, IL‐1β, IL‐18, and TNFα. *n* = 4. ^#^ *p* < 0.05, ^##^ *p* < 0.01, and ^###^ *p* < 0.001 versus the control group; *p < 0.05, **p < 0.01, and ***p < 0.001 versus the D‐gal group; n.s. no statistical difference; one‐way ANOVA.

To investigate whether pyroptosis is implicated in the senescence of hair cells induced by dysfunctional OPA1, we conducted experiments aimed at validating the pertinent inflammatory pathways. Western blotting revealed a significant increase in the expression of inflammatory factors in senescent HEI‐OC1 cells, including NLRP1, NLRP3, caspase 1, IL‐1β, and active forms thereof, and other factors including IL‐18 and TNFα (Figure S3a–i). Furthermore, following the inhibition of L‐OPA1 with MYLS22 or Tyrphostin A9, the levels of the aforementioned inflammatory factors increased significantly, suggesting a notable exacerbation of the inflammatory response (Figure 6a–i). These results imply that the dysregulation of mitochondrial dynamics mediated by dysfunctional OPA1 can trigger inflammatory pathways associated with pyroptosis, potentially playing a critical role as a regulator of the aging of cochlear hair cells.

## 4 DISCUSSION

Here, we determined for the first time that variations in the activity and quantity of the mitochondrial inner membrane fusion protein L‐OPA1 significantly contribute to the occurrence and development of ARHL. Increased OPA1 acetylation levels and excessive cleavage can prompt the dysregulation of mitochondrial dynamics characterized by excessive fission, which may ultimately accelerate the senescence and death of cochlear hair cells via the activation of the pyroptotic inflammatory pathway.

### 4.1 Processing of OPA1: L‐OPA1 and S‐OPA1

Some scholars posit that the two hydrolases, YME1L1 and OMA1, exert distinct effects on OPA1 processing (MacVicar & Langer, 2016), where OMA1 specifically targets the S1 site and YME1L1 targets the S2 site (Ishihara et al., 2006). Since the S1 site of OPA1 is present in all isoforms, whereas the S2 site is present in only half of the OPA1 isoforms, only the activation of OMA1 has the potential to facilitate the complete conversion of L‐OPA1 to S‐OPA1 (MacVicar & Langer, 2016). In this study, we found the upregulation of OMA1 and the downregulation of YME1L1 in senescent HEI‐OC1 cells. A study published in science in 2015 reported that the deletion of YME1L1 in cardiomyocytes did not prevent OPA1 cleavage and the function of mitochondrial respiratory chain. Instead, its deletion even prompted OPA1 cleavage by OMA1, subsequently promoting mitochondrial fission (Wai et al., 2015). Therefore, there is reason to believe that the decline in L‐OPA1 levels observed in ARHL is attributable to the activation of OMA1 rather than YME1L1.

Considering the subcellular localization of the two OPA1 molecules, S‐OPA1 is confined to accumulating within the mitochondrial membrane space due to the cleavage and loss of its membrane‐binding domain. Consequently, only L‐OPA1 but not S‐OPA1 can localize to the inner mitochondrial membrane to exert its principal function of promoting inner membrane fusion (MacVicar & Langer, 2016). Therefore, this study primarily focused on the function and activity of L‐OPA1. However, since the acetylation modification sites exist in both OPA1 molecules and there are no specific structural distinctions between them, it is not feasible to exclusively detect acetylation modifications only on L‐OPA1. Therefore, we were compelled to assess the total OPA1 acetylation activity to substantiate our hypothesis, acknowledging that this is a limitation of our experimental approach.

### 4.2 Diminished L‐OPA1 levels are pivotal to promoting mitochondrial fission in ARHL

Currently, it is widely accepted that the irreversible degeneration of cochlear hair cells constitutes the principal pathological basis of ARHL (He et al., 2019; Keithley, 2020; Liu et al., 2022). Some scholars have reported that OHCs are more susceptible to age‐related degeneration than IHCs (Keithley, 2020), which is consistent with the *in vivo* experimental results in this study. Most studies focused on the connection between mitochondria and hair cell degeneration have been limited to topics such as oxidative stress, mitophagy, and mitochondrial DNA (mtDNA) damage (Chen & Tang, 2014; He, Li, et al., 2021; Kishimoto‐Urata et al., 2022), whereas few studies have focused on mitochondrial dynamics homeostasis. Given that OPA1 is a crucial protein involved in maintaining mitochondrial dynamics, it has been demonstrated that an excessive proteolysis of OPA1 can trigger mitochondrial fragmentation, consequently leading to heart failure in mice (Wai et al., 2015). Another study showed that the stabilization of L‐OPA1 reduced neuronal apoptosis and preserved mitochondrial function to protect neuronal cells from ischemic injury (Lai et al., 2020). Furthermore, it has been found that age‐related reduction of OPA1 is associated with muscle loss in sedentary humans (Tezze et al., 2017). The cited evidence suggests that OPA1‐mediated mitochondrial dysfunction is involved in the pathogenesis of various neurodegenerative diseases.

In this study, we found excessively fragmented mitochondria in the D‐gal‐induced *in vitro* model of hair cell senescence. Furthermore, we also found the presence of fragmented mitochondria exhibiting cristae loss in cochlear hair cells from 24‐month‐old mice. Our findings indicate that these mitochondrial abnormalities may arise from abnormalities in the acetylation activity and quantity of L‐OPA1. These facts further imply that mitochondrial fusion dysfunction dominated by L‐OPA1 may constitute a pivotal factor contributing to the progression of ARHL.

### 4.3 The effect of SIRT3/OPA1 signaling on ARHL

The lysine acetylation of proteins is an important PTM. It has been reported that PTM is involved in the pathogenesis of various neurodegenerative diseases, including Alzheimer's disease, Parkinson's disease, and Huntington's disease (Mattson, 2003; Wang et al., 2020). Approximately 63% of mitochondrial proteins possess lysine acetylation sites (Baeza et al., 2016). As a crucial mitochondrial deacetylase, SIRT3 plays a pivotal role in preserving the regular physiological functions of mitochondrial proteins (Shen et al., 2020). OPA1 is a SIRT3 effector molecule, and recent studies have indicated that the interaction between these two proteins is presumably direct. The study in a cardiac stress model confirmed that SIRT3 directly bound to and deacetylated OPA1 (Samant et al., 2014), and several scholars reported that SIRT3 exhibited the capability to directly deacetylate and regulate OPA1 in HiPSC‐CMs (Wang et al., 2023). One study in cardiomyocytes demonstrated that SIRT3‐dependent deacetylation of OPA1 is currently the sole identified PTM that promotes OPA1 activity (Benigni et al., 2019). However, there have been no studies reporting on the connection between acetylation modifications of OPA1 and cochlear disorders, including hearing loss.

To elucidate the involvement of OPA1 in the occurrence and progression of ARHL, we observed the direct binding of SIRT3 to OPA1 and the inhibition of the SIRT3/OPA1 signaling in senescent HEI‐OC1 cells. This suggested that in addition to the abnormal cleavage of OPA1, the acetylation modification of OPA1 may significantly influence the pathogenesis of ARHL. Subsequently, following the activation of SIRT3 through the administration of kaempferol, we observed a substantial reduction in the level of OPA1 acetylation, accompanied by the significant amelioration of the cellular senescence phenotype. In addition, i*n vivo* observations similarly revealed that the activation of the SIRT3/OPA1 signaling significantly reduced degeneration and disruption of mitochondrial morphology or cristae structures in cochlear hair cells and improved auditory function. These findings suggest that the SIRT3/OPA1 signaling plays a pivotal role in the aging and degeneration of cochlear hair cells.

Furthermore, some researchers have reported a decrease in the expression of OPA1 following SIRT3 knockout in human‐induced pluripotent stem cells, whereas OPA1 expression increased upon SIRT3 activation (Wang et al., 2023). This suggests that the expression of OPA1 is also regulated by SIRT3 alterations, which implies that the interaction between OPA1 and SIRT3 extends beyond acetylation and warrants further research focused on the specific regulatory mechanisms (R. Wang et al., 2023).

### 4.4 Inflammatory pathways associated with pyroptosis and ARHL

Inflammation is a crucial hallmark of the aging process (López‐Otín et al., 2023), and chronic inflammation significantly impacts cochlear aging (He et al., 2020; Watson et al., 2017). Some groups have detected elevated levels of pro‐inflammatory factors including TNFα, IL‐1β, and IL‐6 in the scala tympani of the cochlea in ARHL mice. These findings imply the presence of chronic inflammation in ARHL (Menardo et al., 2012). Another single‐cell transcriptome sequencing study in the cochlea of aging C57BL/6J mice has revealed that escalated inflammation is one of the key features across the timeline of cochlear aging (Sun et al., 2023). Certain scholars have identified that the disruption of mitochondrial cristae regulators, such as OPA1, can activate the IFN‐I inflammatory response, mechanistically linking mitochondrial cristae disorder and inflammation (He et al., 2022). In this study, we found that the NLRP1/3 inflammasome was activated and that levels of caspase 1, IL‐1β, and pro‐inflammatory factors such as IL‐18 and TNFα were increased following the dysregulation of mitochondrial dynamics induced by inhibiting L‐OPA1, suggesting that such dysregulation can lead to the activation of the inflammatory response. The resultant inflammation, in turn, damages cochlear structures, thereby accelerating the senescence and degeneration of cochlear hair cells. Further studies focused on other aspects of inflammation‐mediated pyroptosis, including the expression of GSDMD (Shi et al., 2017) and the observation of cell membrane morphology, are warranted.

## 5 CONCLUSION

During the progression of ARHL, the abnormal OPA1 molecular function resulting from excessive cleavage of L‐OPA1 and the increase in OPA1 acetylation owing to reductions in SIRT3 levels mediates the dysregulation of mitochondrial dynamics within cochlear hair cells. These changes can activate pyroptosis‐related inflammatory pathways, consequently expediting the senescence of cochlear hair cells. In contrast, the activation of the SIRT3/OPA1 signaling can mitigate the age‐related degeneration of cochlear hair cells and reduce hearing loss in aged mice. The findings of this study underscore the significant role that abnormalities in the quantity and activity of OPA1 play in the occurrence and development of ARHL.

## AUTHOR CONTRIBUTIONS

All of the authors listed made substantial contributions to the manuscript and qualify for authorship. Mingliang Xiang and Bin Ye contributed to conception and design of the work. Andi Zhang and Yi Pan conducted the experiments, analysis, and interpretation of data. Hao Wang, Tianyuan Zou, Dongye Guo, Yilin Shen, Peilin Ji, Weiyi Huang, Jichang Wu, and Rui Ding contributed to acquisition of data and development of methodology. Andi Zhang wrote the manuscript. Mingliang Xiang, Bin Ye, Qing Wen, Quan Wang and Haixia Hu contributed to the revision of the manuscript. Mingliang Xiang and Bin Ye contributed to funding acquisition and project administration. All authors have read and approved the final manuscript.

## FUNDING INFORMATION

The study was supported by grants from the National Natural Science Foundation of China (grant No. 82101212, 82101209, 82301296, 82301297) and Science and Technology Commission of Shanghai Municipality (grant No. 23ZR1440200, 21ZR1440200, SHDC2020CR1044B‐003).

## CONFLICT OF INTEREST STATEMENT

All authors declare that they have no known competing financial interests or personal relationships that could have appeared to influence the work reported in this paper.

## Supporting information


**Fig. S1.** Mitochondrial dysfunction is present in HEI‐OC1 cells following D‐gal‐induced senescence. (a) Cell viability was measured after treatment with different concentrations of D‐gal for 6, 24, and 48 h. The IC_50_ was calculated using drug fitting curve analyses. (b–c) Statistical results pertaining to SA‐β‐gal staining and the proportion of senescence‐positive HEI‐OC1 cells after D‐gal treatment. As the concentration of D‐gal increased, the proportion of senescent hair cells gradually increased and the positive staining (Blue) deepened. Scale bar: 20 μm. *n* = 5. (d, e) Western blotting revealed the expression of senescence marker proteins in senescent HEI‐OC1 cells, including increased p‐P53/P53 and P21 levels. *n* = 5. (f) Analyses of ATP content within senescent HEI‐OC1 cells, which indicated that ATP production was significantly reduced. *n* = 6. (g, h) ROS content and mean fluorescence intensity (MFI) levels in senescent HEI‐OC1 cells were measured by flow cytometry in the FITC channel, showing that ROS accumulation increased significantly in senescent cells. *n* = 3. (i, j) MtROS content and MFI levels in senescent HEI‐OC1 cells were measured by flow cytometry in the PE channel, showing that mtROS accumulation increased significantly in senescent cells. *n* = 3. *p < 0.05, **p < 0.01, and ***p < 0.001 versus the control group; n.s. no statistical difference; one‐way ANOVA.

**Fig. S2.** L‐OPA1 inhibition exacerbates mitochondrial fragmentation in senescent HEI‐OC1 cells. (a) CCK‐8 assay revealed that either MYLS22 or Tryphostin A9 could exacerbate the damage and death of senescent HEI‐OC1 cells. (b, c) Statistical analyses of Western blotting results focused on the expression of mitochondrial dynamics‐related proteins revealed that both small molecule compounds were able to inhibit the expression of L‐OPA1. *n* = 5. (d) Mitotracker staining indicated that the mitochondrial fragmentation in senescent cells was aggravated after inhibiting L‐OPA1 using these two compounds respectively. Scale bar: 5 μm. ^#^ *p* < 0.05, ^##^ *p* < 0.01, and ^###^ *p* < 0.001 versus the control group; *p < 0.05, **p < 0.01, and ***p < 0.001 versus the D‐gal group; n.s. no statistical difference; one‐way ANOVA.

**Fig. S3.** Senescent HEI‐OC1 cells exhibit activation of inflammatory pathways. (a) Western blotting revealed the expression of inflammatory pathway molecules in senescent HEI‐OC1 cells after D‐gal treatment. (b–i) Statistical analyses of western blotting results indicated a significant increase in the expression of inflammatory factors in D‐gal‐induced senescent HEI‐OC1 cells, including NLRP1/3, caspase 1, IL‐1β, IL‐18, and TNFα. *n* = 5. *p < 0.05, **p < 0.01, and ***p < 0.001 versus the control group; n.s. no statistical difference; one‐way ANOVA.

## Data Availability

The source data for each figure in this study are available from the corresponding author on reasonable request.
