# Supplementary figures and images for: Excessive processing and acetylation of OPA1 aggravate age‐related hearing loss via the dysregulation of mitochondrial dynamics
Source: Aging Cell. 2024 Jan 24;23(4):e14091. doi: 10.1111/acel.14091 (PMC11019136; doi:10.1111/acel.14091)

Figure S1

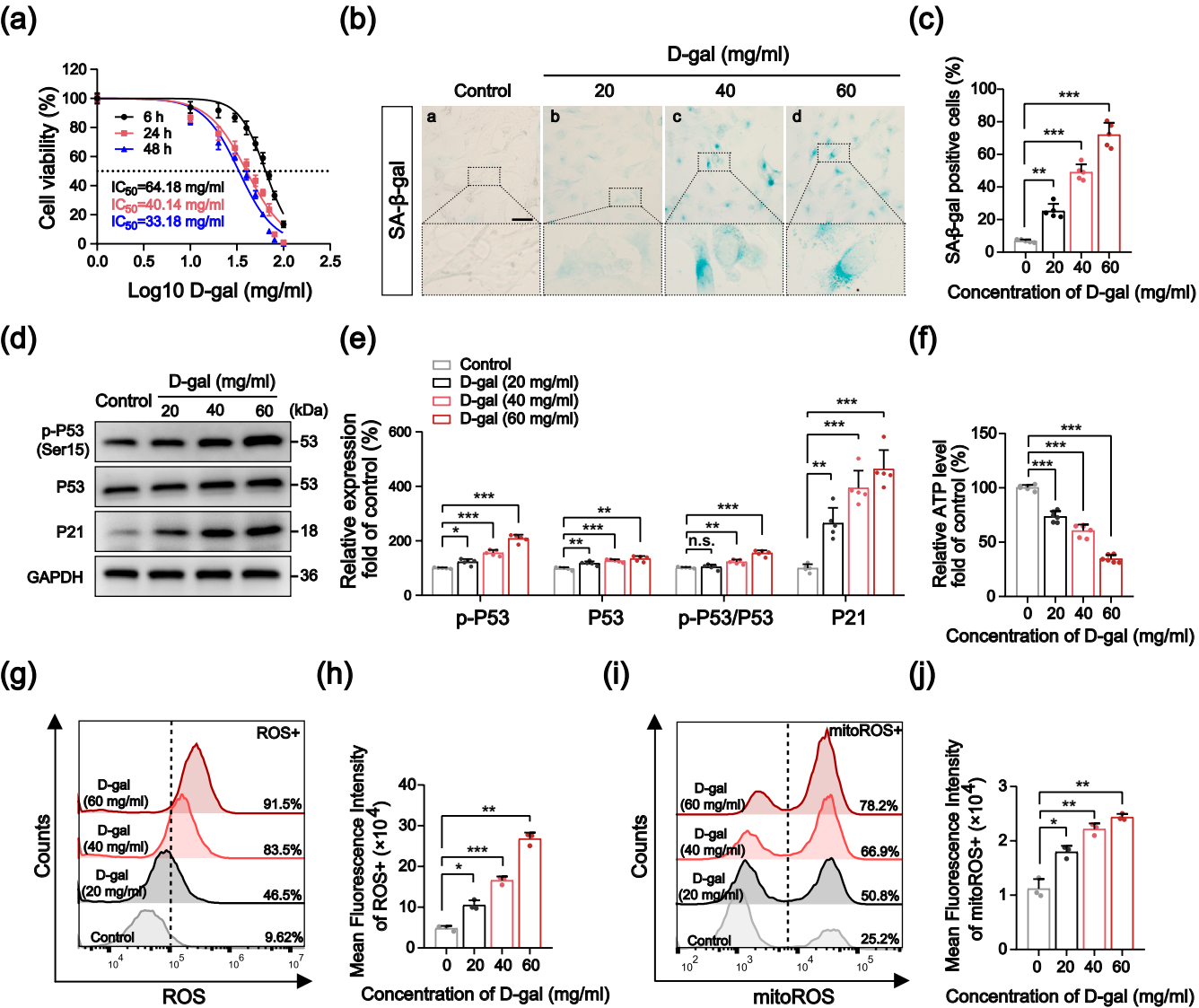

Supplement: Supplementary file 1 — Fig. S1. Mitochondrial dysfunction is present in HEI‐OC1 cells following D‐gal‐induced senescence. (a) Cell viability was measured after treatment with different concentrations of D‐gal for 6, 24, and 48 h. The IC50 was calculated using drug fitting curve analyses. (b–c) Statistical results pertaining to SA‐β‐gal staining and the proportion of senescence‐positive HEI‐OC1 cells after D‐gal treatment. As the concentration of D‐gal increased, the proportion of senescent hair cells gradually increased and the positive staining (Blue) deepened. Scale bar: 20 μm. n = 5. (d, e) Western blotting revealed the expression of senescence marker proteins in senescent HEI‐OC1 cells, including increased p‐P53/P53 and P21 levels. n = 5. (f) Analyses of ATP content within senescent HEI‐OC1 cells, which indicated that ATP production was significantly reduced. n = 6. (g, h) ROS content and mean fluorescence intensity (MFI) levels in senescent HEI‐OC1 cells were measured by flow cytometry in the FITC channel, showing that ROS accumulation increased significantly in senescent cells. n = 3. (i, j) MtROS content and MFI levels in senescent HEI‐OC1 cells were measured by flow cytometry in the PE channel, showing that mtROS accumulation increased significantly in senescent cells. n = 3. *p < 0.05, **p < 0.01, and ***p < 0.001 versus the control group; n.s. no statistical difference; one‐way ANOVA. [file ACEL-23-e14091-s002.pdf]

**Figure S2****(a)**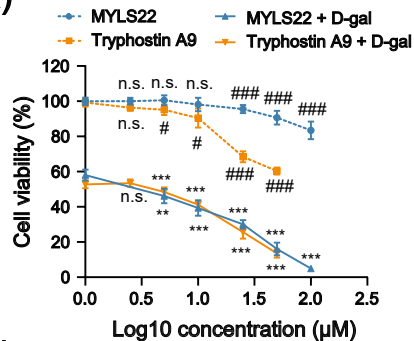**(b)**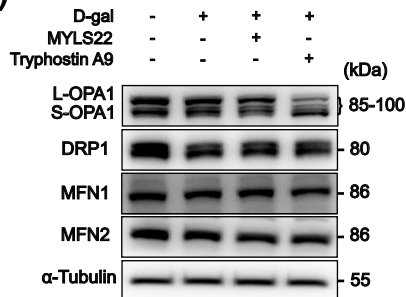**(d)**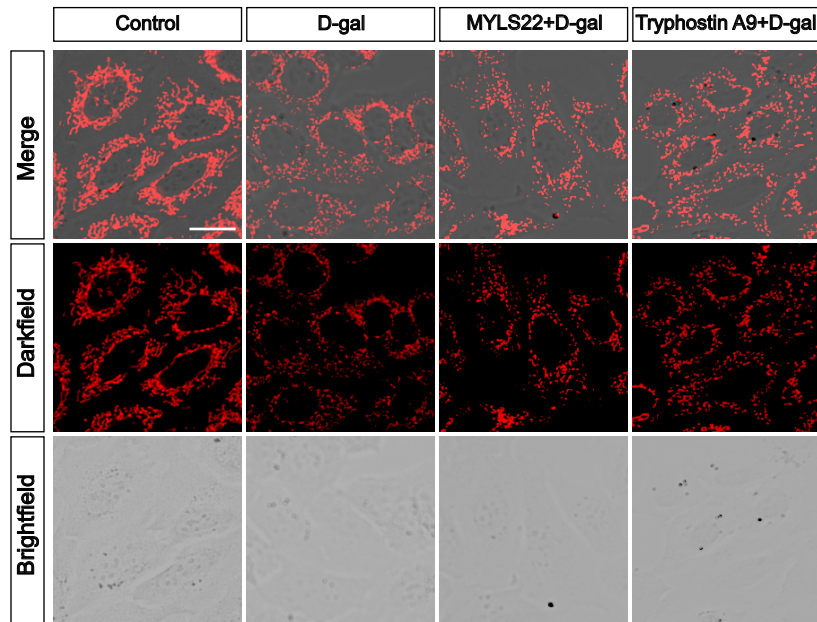**(c)**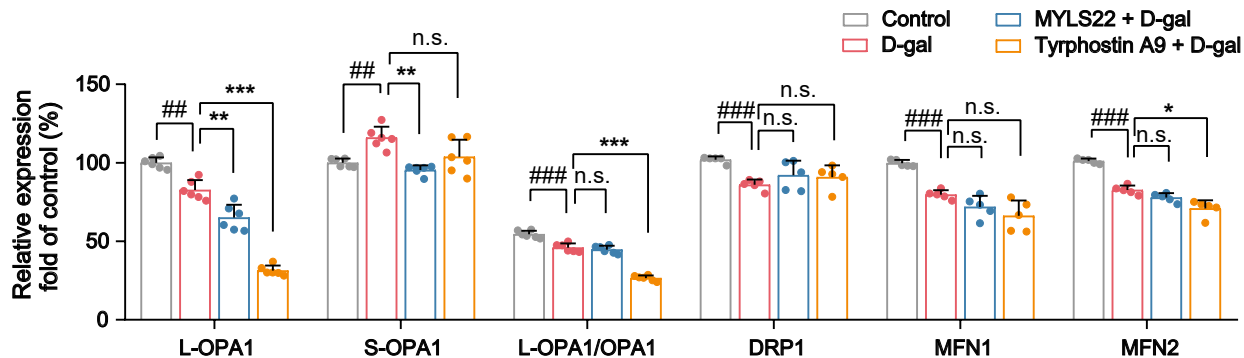

Supplement: Supplementary file 2 — Fig. S2. L‐OPA1 inhibition exacerbates mitochondrial fragmentation in senescent HEI‐OC1 cells. (a) CCK‐8 assay revealed that either MYLS22 or Tryphostin A9 could exacerbate the damage and death of senescent HEI‐OC1 cells. (b, c) Statistical analyses of Western blotting results focused on the expression of mitochondrial dynamics‐related proteins revealed that both small molecule compounds were able to inhibit the expression of L‐OPA1. n = 5. (d) Mitotracker staining indicated that the mitochondrial fragmentation in senescent cells was aggravated after inhibiting L‐OPA1 using these two compounds respectively. Scale bar: 5 μm. # p < 0.05, ## p < 0.01, and ### p < 0.001 versus the control group; *p < 0.05, **p < 0.01, and ***p < 0.001 versus the D‐gal group; n.s. no statistical difference; one‐way ANOVA. [file ACEL-23-e14091-s001.pdf]

**Figure S3****(a)**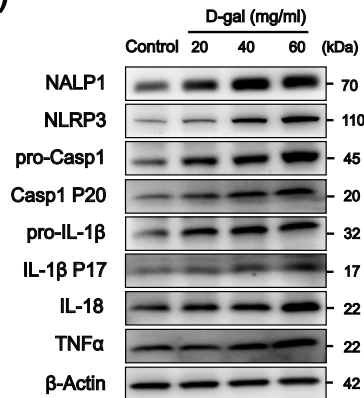**(b)**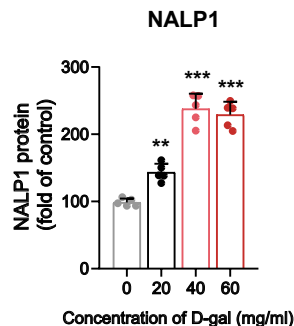**(c)**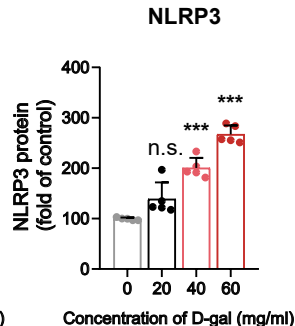**(d)**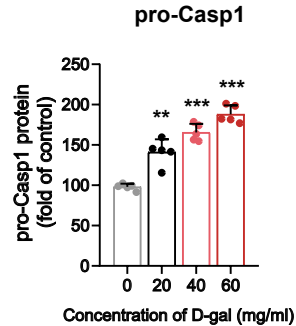**(e)**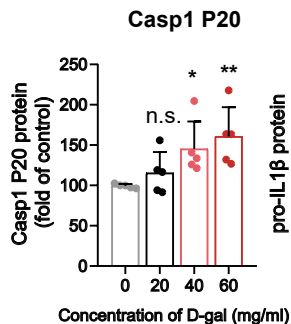**(f)**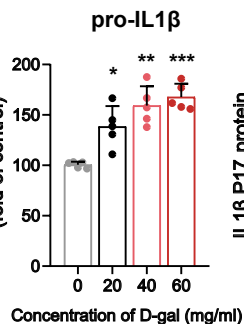**(g)**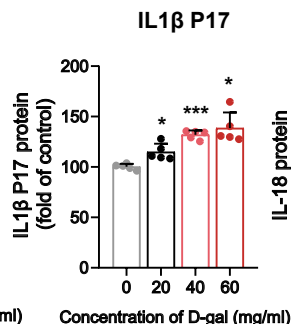**(h)**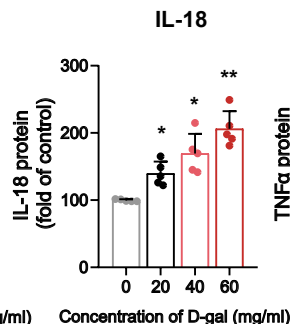**(i)**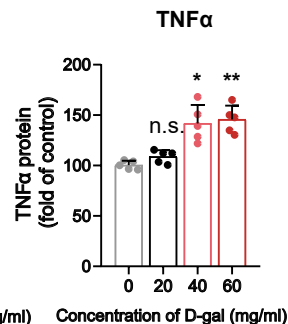

Supplement: Supplementary file 3 — Fig. S3. Senescent HEI‐OC1 cells exhibit activation of inflammatory pathways. (a) Western blotting revealed the expression of inflammatory pathway molecules in senescent HEI‐OC1 cells after D‐gal treatment. (b–i) Statistical analyses of western blotting results indicated a significant increase in the expression of inflammatory factors in D‐gal‐induced senescent HEI‐OC1 cells, including NLRP1/3, caspase 1, IL‐1β, IL‐18, and TNFα. n = 5. *p < 0.05, **p < 0.01, and ***p < 0.001 versus the control group; n.s. no statistical difference; one‐way ANOVA. [file ACEL-23-e14091-s003.pdf]
